# PRECARITY IN OLD AGE IN AMERICA

**DOI:** 10.1093/geroni/igz038.2183

**Published:** 2019-11-08

**Authors:** Larry Polivka

**Affiliations:** Florida State University, Tallahassee, Florida, United States

## Abstract

This presentation will analyze the growing precarity of life for older Americans emanating from austerity budgets and privatization of public services. As governments at every level continue to look for ways to reduce social and health spending and cut taxes they are also pursuing privatization through contracts with for-profit firms to provide regulated social and health services. The presentation will discuss how these trends toward greater precarity in old age relate to Streecks’ concept of the Consolidation State in Western neoliberal political economies. The Consolidation State serve the interest of capital, especially finance capital by implementing austerity budgets, preserving low taxes on wealth, prioritizing the financing of deficits/debt and privatizing public services and assets. Such policies put health care, long term care and other programs supporting older persons in jeopardy, creating a glide path toward the extension of precarious employment into a precarious retirement for millions of older people.

